# Supplementary material for: Microbial secondary succession in soil microcosms of a desert oasis in the Cuatro Cienegas Basin, Mexico
Source: PeerJ. 2013 Mar 5;1:e47. doi: 10.7717/peerj.47 (PMC3628611; doi:10.7717/peerj.47)
Supplement: Table S1 — PCR primers conditions for the genes amplified. All the reactions include a final extension of 10 min at 72 °C. [file peerj-01-47-s003.pdf]

Table S1. PCR primers conditions for the genes amplified. All the reactions include a final extension of 10 min at 72°C.

Gene	Primer name	Primer sequence	Fragment size (pb)	Thermocycling conditions		Reference
				Initial denaturing step	Amplification cycles	
16S rRNA	*F27*	5’ AGA GTT TGA TCM TGG CTC AG 3’	~1500	95°C<br>10 min	95°C 1 min<br>52°C 2 min 30 cycles<br>72°C 3 min	Wilmotte *et al*., 1993
	R1492	5’ TAC GGY TAC CTT GTT ACG ACT T 3’				

Wilmotte,A., Van der Auwera,G.,De Wachter, R.1993. Structure of the 16 S ribosomal RNA of the thermophilic cyanobacterium chlorogloeopsis HTF (‘mastigocladus laminosus HTF’) strain PCC7518, and phylogenetic analysis.FEBS letters 317(1-2):96-100.
