# Supplementary material for: Microbial secondary succession in soil microcosms of a desert oasis in the Cuatro Cienegas Basin, Mexico
Source: PeerJ. 2013 Mar 5;1:e47. doi: 10.7717/peerj.47 (PMC3628611; doi:10.7717/peerj.47)
Supplement: Table S2 — PCR reaction mix for the genes amplified. [file peerj-01-47-s004.pdf]

TableS2. PCR reaction mix for the genes amplified.

Reactive/Gene	16S rRNA
10X PCR Buffer	5µl
$MgCl_2$	1.0 µM
dNTPs	0.2 mM
Platinum Taq DNA High Fidelity Polymerase (Invitrogen)	1 unit
Primers (of each one)	0.2 µM
DMSO	5%
BSA	0.05 µg/µl
Triton	-
