# Supplementary material for: Microbial secondary succession in soil microcosms of a desert oasis in the Cuatro Cienegas Basin, Mexico
Source: PeerJ. 2013 Mar 5;1:e47. doi: 10.7717/peerj.47 (PMC3628611; doi:10.7717/peerj.47)
Supplement: Table S3 — Barcode sequences used for pooled amplicon 454 Pyrosequencing run. Linker primers were all identical (TTGACGGGGGCCCGCAC). [file peerj-01-47-s005.pdf]

Table S3: Barcode sequences used for pooled amplicon 454 Pyrosequencing run. Linker primers were all identical (TTGACGGGGGCCCGCAC).

Sample	Barcode	Total # Seqs.
Dry lagoon		
Before	AGATCGCT	32633
3 months	AGATCTGT	39705
6 months	AGATGATT	40860
9 months	AGATGGAT	16377
12 months	AGATGTCT	30211
Undisturbed	AGATTAGT	36394
River		
Before	AGATTCTT	39427
3 months	AGCAACGT	41308
6 months	AGCAAGTT	40481
9 months	AGCACAAT	59130
12 months	AGCACGGT	27827
Undisturbed	AGCAGCAT	32006
