# Supplementary material for: Microbial secondary succession in soil microcosms of a desert oasis in the Cuatro Cienegas Basin, Mexico
Source: PeerJ. 2013 Mar 5;1:e47. doi: 10.7717/peerj.47 (PMC3628611; doi:10.7717/peerj.47)
Supplement: Table S4 — Distribution of the most abundant bacterial families. The relative abundance of bacterial families with more than 1% in at least two libraries in each of the sampling dates and sites. Values represent the percentage of each group in the respective library. [file peerj-01-47-s006.pdf]

Table S4. Distribution of the most abundant bacterial Families. The relative abundance of bacterial families with more than 1% in at least two libraries in each of the sampling dates and sites. Values represent the percentage of each group in the respective library.

	Dry lagoon						River					
	Before sterilization	3 months	6 months	9 months	12 months	Undisturbed soil	Before sterilization	3 months	6 months	9 months	12 months	Undisturbed soil
**Acidobacteriaceae**	24.48	0.01	17.90	13.07	21.66	20.80	45.75	14.21	21.05	28.81	23.33	32.12
**Anaerolineaceae**	0.60	0.00	0.77	1.91	0.49	3.89	0.93	0.00	1.08	1.63	0.81	5.35
**Bacillaceae**	0.07	1.26	0.23	0.01	0.09	0.00	0.24	3.36	0.10	0.02	0.16	0.04
**BD2-11 Gemmatimonadetes**	0.11	0.00	0.37	0.50	1.04	0.53	0.50	0.00	1.21	2.45	1.44	0.58
**Caldilineaceae**	0.08	0.00	0.13	0.57	0.10	1.03	0.33	0.00	0.57	0.85	0.29	3.16
**Chitinophagaceae**	0.22	0.00	1.59	2.42	3.99	0.64	6.94	0.00	3.78	5.30	4.42	0.56
**Cyclobacteriaceae**	3.43	0.00	0.10	0.36	0.65	0.01	0.38	0.00	0.25	0.57	1.00	0.10
**Desulfobacteraceae**	0.00	0.00	0.01	0.27	0.00	1.79	0.52	0.04	2.28	1.60	0.10	3.14
**Ectothiorhodospiraceae**	0.05	0.00	0.23	0.08	0.37	0.18	0.39	0.00	4.25	4.51	0.73	0.64
**Enterobacteriaceae**	0.10	0.00	0.02	0.00	0.04	0.06	0.05	7.81	0.04	0.12	0.15	0.09
**Flavobacteriaceae**	0.30	0.00	0.07	2.69	1.18	0.25	0.69	0.00	0.10	2.89	1.36	0.20
**GIF3 Chloroflexi**	0.00	0.00	0.00	0.00	0.00	0.00	0.02	6.17	0.14	0.07	0.21	0.51
**Halanaerobiaceae**	0.00	0.00	0.00	0.00	0.00	2.26	0.06	0.00	0.22	0.81	0.18	2.14
**Halomonadaceae**	0.02	0.02	0.23	0.48	3.62	0.05	0.61	0.00	0.19	0.10	4.15	0.12
**Holophagaceae**	0.37	0.00	0.35	0.39	1.48	0.57	6.49	20.16	10.02	4.10	6.01	7.03
**Idiomarinaceae**	0.08	0.00	0.06	0.44	1.16	0.08	0.24	0.00	0.03	0.06	1.28	0.00
**Iii1-8 Holophagales**	0.38	0.00	1.05	0.40	0.03	0.02	1.18	0.00	1.93	2.38	0.66	0.71
**LCP-89 Deferribacteres**	0.00	0.00	0.00	0.00	0.00	0.00	0.87	1.08	1.48	0.16	0.30	1.51
**Listeriaceae**	0.00	0.00	0.00	0.00	0.00	0.00	0.00	5.05	0.00	0.00	0.01	0.00

**MBMPE71 Planctomycetes**	0.00	0.00	0.00	0.00	0.00	0.01	0.01	4.62	0.03	0.01	0.25	0.13
**Moraxellaceae**	2.33	18.01	1.68	0.68	0.57	1.53	1.89	1.61	2.17	2.07	0.52	0.80
**OP11 unclasified**	0.00	0.00	0.00	0.00	0.00	0.03	0.02	0.00	0.41	1.86	0.02	0.82
**Phycisphaeraceae**	0.50	0.00	0.11	0.00	0.01	0.01	0.04	0.00	1.05	1.51	1.07	0.06
**Piscirickettsiaceae**	0.01	0.00	0.02	1.95	1.41	0.00	1.85	0.00	0.06	0.03	1.87	0.02
**Pseudomonadaceae**	0.63	5.32	0.25	2.17	2.21	0.03	1.23	0.80	0.41	0.55	2.05	0.12
**Puniceicoccaceae**	0.05	0.00	0.00	1.70	1.05	0.33	0.11	0.00	0.21	0.35	1.08	0.51
**RB25 Acidobacteria Holophag**	0.06	0.01	0.10	0.01	0.13	0.54	2.18	10.99	7.21	1.80	14.13	2.59
**SHA-26 Chloroflexi**	0.01	0.00	0.03	0.02	0.04	0.00	0.43	2.20	0.41	1.22	2.08	1.40
**SJA-36 Acidobacteria Holopha**	0.01	0.00	0.01	0.00	0.02	0.00	3.63	3.94	6.37	1.73	1.76	3.70
**Sporolactobacillaceae**	0.00	0.00	0.00	0.00	0.00	0.02	0.07	2.12	0.01	0.00	0.05	0.00
**Streptococcaceae**	0.13	7.97	0.07	0.32	0.00	0.00	0.02	5.82	0.03	0.00	0.00	0.00
**TA06**	0.00	0.00	0.00	0.00	0.00	0.00	0.00	2.97	0.08	0.00	0.51	0.06
**TM6 unclassified**	0.46	0.00	0.13	4.53	0.51	0.80	0.89	0.02	0.94	3.60	0.38	1.09
**TM7 unclasified**	0.23	0.00	0.05	3.65	0.42	0.22	0.39	0.00	0.39	1.18	0.52	0.43
**Trueperaceae**	2.77	0.33	6.03	2.26	0.69	0.47	0.38	0.00	5.98	5.42	0.85	0.20
**Vibrionaceae**	0.01	0.00	0.07	0.20	2.13	0.00	0.10	0.00	0.05	0.05	2.70	0.01
**Xanthomonadaceae**	0.98	0.36	2.71	1.45	0.78	0.07	1.98	0.09	4.55	3.23	0.87	0.15
**unclassified**	33.59	12.01	33.56	26.63	18.21	30.55	0.00	0.00	0.00	0.00	0.00	0.00
**Unclassified Acidobacteria**	14.54	30.52	17.86	9.06	18.92	9.18	0.00	0.00	0.00	0.00	0.00	0.00
**Unclassified Halophaga**	4.56	0.01	4.75	1.32	0.44	2.25	0.00	0.00	0.00	0.00	0.00	0.00
**Alcaligenaceae**	0.00	6.55	0.00	0.30	0.02	0.00	0.03	0.00	0.00	0.00	0.01	0.00
**unclassified deltaproteobacter**	0.26	5.73	0.06	0.20	0.09	0.32	0.00	0.00	0.00	0.00	0.00	0.00
**Micrococcaceae**	0.11	3.05	0.09	0.25	0.02	0.13	0.13	0.14	0.44	0.38	0.28	0.44
**unclassified gammaproteobact**	0.62	1.06	1.24	1.89	2.67	1.01	0.00	0.00	0.00	0.00	0.00	0.00
**unclassified proteobacteria**	0.72	0.47	1.13	1.17	1.13	1.87	0.00	0.00	0.00	0.00	0.00	0.00
**Unclassified Chloroflexi**	0.16	0.00	0.30	2.11	0.32	4.09	0.00	0.00	0.00	0.00	0.00	0
