# Supplementary material for: Microbial secondary succession in soil microcosms of a desert oasis in the Cuatro Cienegas Basin, Mexico
Source: PeerJ. 2013 Mar 5;1:e47. doi: 10.7717/peerj.47 (PMC3628611; doi:10.7717/peerj.47)
Supplement: Table S5 — Meteorological data during the experimental period. Meteorological data from Rancho PRONATURA proportionated by Instituto Nacional de Investigaciones Forestales, Agrícolas y Pecuarias (INIFAP). [file peerj-01-47-s007.pdf]

Table S5. Meteorological data during the experimental period. Meteorological data from Rancho PRONATURA proportionated by Instituto Nacional de Investigaciones Forestales, Agrícolas y Pecuarias (*INIFAP*).

Date	Total Precipitation (mm)	Maximun Temperature (°C)	Minimun Temperatur e (°C)	Average (°C)	Relative humidity (%)
1-feb-2007	0	25.34	5.68	15.34	50.47
1-mar-2007	0	28.98	11.01	20.34	46.39
1-abr-2007	0.4	30.75	12.32	22.03	44.83
1-may-2007	20.6	33.19	18.09	25.79	52.78
1-jun-2007	63.4	35.5	20.52	27.76	54.79
1-jul-2007	13	33.39	20.94	26.85	62.54
1-ago-2007	22.6	33.37	22.61	27.65	57.3
1-sep-2007	19	32.66	19.85	26.05	63.19
1-oct-2007	0.4	31.66	12.83	22.57	48.3
1-nov-2007	14	26.1	7.2	16.81	54.89
1-dic-2007	0	25.39	3.62	13.86	40.36
1-ene-2008	0	21.69	2.05	11.63	44.51
1-feb-2008	0.8	28.8	4.81	17.07	29.92
